# The New Buildings at St. Bartholomew's Hospital

**Published:** 1907-11-02

**Authors:** 


### THE NEW BUILDINGS AT ST. BARTHOLOMEW'S HOSPITAL.

BY OUR SPECIAL CORRESPONDENT.

On the evening of Wednesday, October 24, the medical staff of St. Bartholomew's Hospital gave a conversazione to view the new out-patient and special department block. The foundation-stone of these buildings was laid by the King in July 1904, and they were formally opened by the Prince of Wales in July of the present year. They consist of three parts: a front and a back block, containing the dispensary, the kitchens, the chemical laboratory, and accommodation for the resident staff; and a central or main block, by far the largest of the three, devoted to the reception and treatment of out-patients. This latter is of four stories. On the ground floor is a waiting-hall, 120 feet in length, for the patients; and opening out of this are two small operating-rooms and a number of consulting-rooms for the casualty officers. Here the majority of the cases will be treated, only the more serious being passed on to the assistant physicians' or assistant surgeons' department on the first floor. There are also ten "surgery beds" on this floor for the reception of casualty cases not requiring admission to the hospital for any length of time.

The second and third floors are given up to the special departments for the treatment of diseases of the eye, ear, throat, skin, etc. On the fourth floor is a clinical lecture theatre. The systems of ventilation and heating are of the latest, the walls are everywhere lined with glazed tiles; there are a round dozen of lifts, and telephones innumerable. It is impossible for those who have not seen them to realise from a bald description the size and completeness of these new buildings. In this respect they stand without rivals among British hospitals, and are likely to remain so for a long time to come.

### The Exhibition of Specimens.

The exhibition of pathological specimens included a comparative novelty in the form of some coloured microphotographs produced by the Sanger-Shepherd process. The photographs of red blood corpuscles containing malaria parasites, and of sections of a melanotic sarcoma and of a thyroid tumour were exceptionally good. Colour photography will be of the greatest value in medicine, especially for teaching purposes, for the ordinary microphotograph is a most unsatisfactory thing. The specimens shown at St. Bartholomew's were transparent positives on glass; from these prints can be taken by the Sanger-Shepherd process, though not, as yet, by the more recent Lumière process. These transparencies make excellent lantern-slides. In another department there was to be seen an improved form of electric centrifuge, brought out by Messrs. Maw. On rotation the tubes containing the material to be centrifugalised fit into the under surface of a horizontal disc of heavy metal. On turning off the current this disc continues to rotate with a free-wheel action, until it very gradually comes to rest, instead of stopping with a sudden jerk, which is the fault with most power-driven centrifuges. This principle has been applied to a water-driven machine, but we have never seen it used for an electric centrifuge.

### Sir Oliver Lodge's Experiments.

Finally, to mention only one other out of many interesting exhibits, some striking photographs were shown in illustration of Sir Oliver Lodge's experiments on the use of electricity in agriculture. He finds that, if a system of electrically-charged wires be stretched on insulated supports at a height of about 15 feet above a field, the crops sown there will grow much more rapidly and vigorously than would otherwise be tbe case; and he presumes that this effect is in some way due to the charge suspended above them. The photographs showed the exuberant growth of an ordinary hedge and of some wheat after treatment in this way.

We must congratulate the authorities of St. Bartholomew's Hospital not only on the possession of a magnificent out-patient department, but also on having already met the cost of this building. By so doing they have departed from the usual methods of hospital economy, where the custom is not to pay the piper until long after the tune has ended. We would also congratulate the future students of St. Bartholomew's, of whom we are almost tempted to be envious, for their opportunities will be unique.

